# Broadening the color palette of optical skyrmions

**DOI:** 10.1038/s41377-026-02466-4

**Published:** 2026-09-08

**Authors:** Mingjian Cheng, Andrew Forbes

**Affiliations:** 1 School of Physics, Xidian University, Xi’an, China; 2 School of Physics, University of the Witwatersrand, Johannesburg, South Africa

**Keywords:** Optical materials and structures, Micro-optics

## Abstract

Optical skyrmions have been demonstrated across many wavelengths, but so far only one at a time for a monochrome color palette. Now they are reported as broadband topologies across the entire visible spectrum, heralding white light skyrmions on-chip.

Topology is found everywhere in Nature, from galactic to nano scales and is considered a global property of a system that is agnostic to its specific underlying features, even if important physics remains embedded in these features. It is in this sense that donuts and coffee mugs are topologically equivalent yet very distinct in how we perceive and use them. The point is acute when creating topologies from waves, their usefulness highly related to how the wave is constructed. Long wavelength topological water waves are ideal for controlling the floating motion of macroscopic objects^1^, acoustic topologies can shape and control micro-defects in matter^2^, telecom wavelength topologies can be passed down optical fiber^3^ and near-infrared topologies suitable for probing biological matter^4^. An area of immediate relevance to photonics is optical topologies in the form of skyrmions^5,6^, now with a sophisticated toolkit that has seen them recently advanced as topologically evolving spatial fields^7^, space-time light^8^ and quantum states^9–11^. The wavelength coverage is across an enormous spectral range with some wavelength switching possible^12^, but so far limited to a narrow a priori choice because the creation mechanisms have been based on resonant processes that restrict wavelength tuneability and range.

Reporting in *eLight*, Yuanfeng Liu and co-workers demonstrate the first on-chip generation of broadband optical Skyrmions^13^, as illustrated in Fig. 1, using a single self-assembled ferroelectric spherulite. The device is a micron-scale dome in which polar rod-like molecules are arranged azimuthally, giving the crystal a circular anisotropy described by different refractive indices along the radial and azimuthal directions. When a right-circularly polarized Gaussian beam enters from the substrate side, the thin outer region leaves the polarization largely unchanged and bends this component towards the focus. The thicker central region acts as a spatially varying waveplate, where the radial and azimuthal field components acquire a relative phase delay. At the correct setting (a delay of π), the light flips handedness and gains orbital angular momentum through spin–orbit coupling. As a result, the central part of the beam is converted into a left-circular vortex with topological charge –2, while the unconverted right-circular component remains unchanged. These two orthogonally polarized modes overlap at the focus to produce a Stokes-vector texture whose local polarization varies across the beam. This texture covers the Poincaré sphere twice, giving a skyrmion number close to –2, while reversing the incident circular polarization reverses the sign of the topology. In effect, the spherulite performs polarization conversion, vortex generation and focusing in one continuous, non-resonant optical element^14^, now exploited for topological control.

**Fig. 1:**
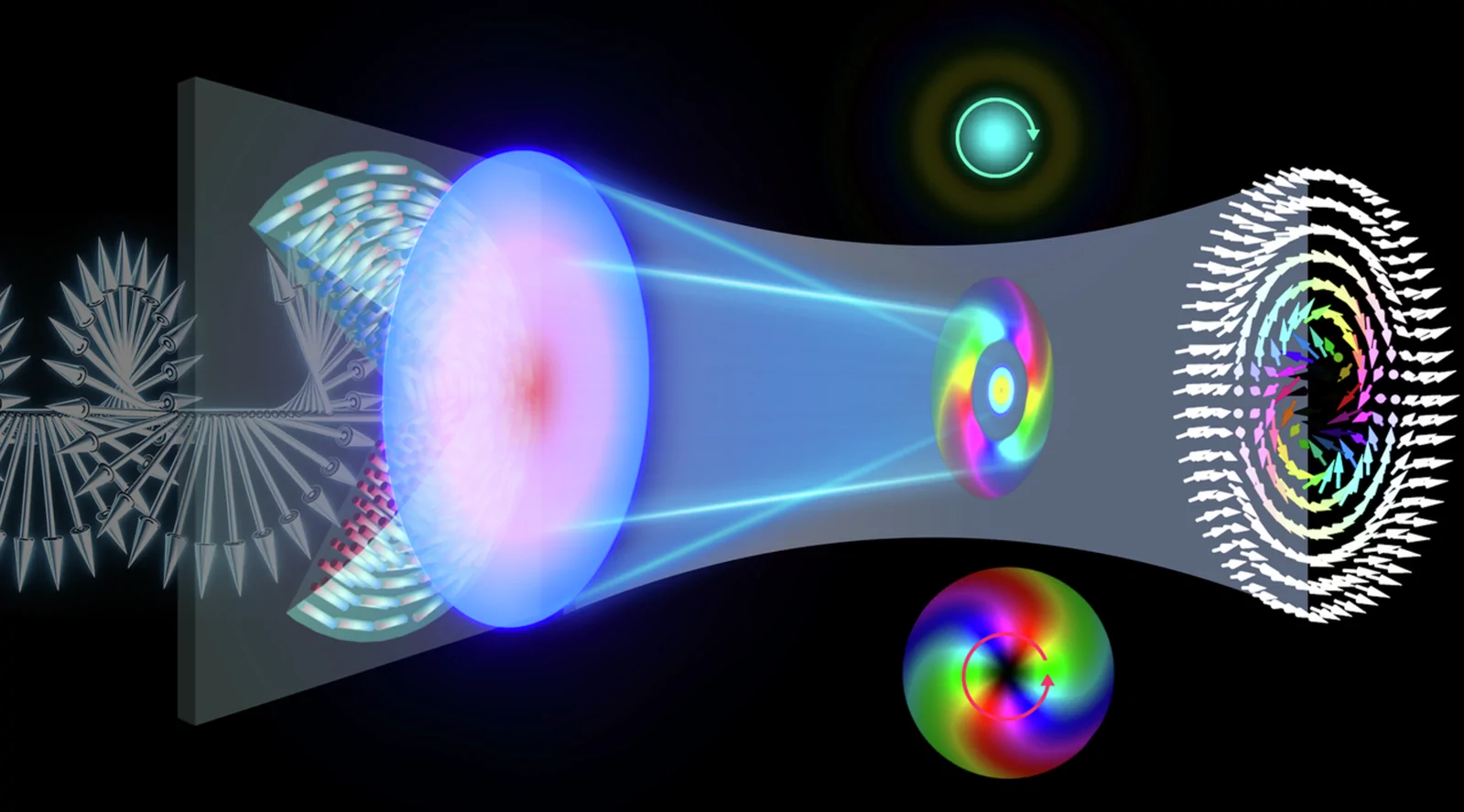
**Broadband skyrmions from a self-assembled ferroelectric spherulite**. A right circularly polarized Gaussian beam is focused through an on-chip ferroelectric spherulite with an azimuthally ordered molecular structure that provides a spatially varying optical anisotropy. The outer region of the microcrystal mainly focuses the unconverted spin component, whereas the thicker central region acts as a geometric waveplate, converting part of the incident light into an opposite-handed optical vortex through spin–orbit coupling. The interference between the fundamental spin component and the generated vortex produces a spatially varying Stokes-vector texture at the focus, forming optical skyrmions across multiple wavelengths

The key question is whether such a self-assembled element is tolerant enough to work across the visible spectrum. The authors show that it is. Because the spherulites form with an approximately fixed contact angle of 35°, their height scales predictably with radius. Simulations reveal a broad design window for high-quality skyrmions across the visible spectrum. The weak dispersion of the material, with nearly constant birefringence across the visible range, further supports broadband operation. Guided by this result, the team fabricated spherulites with a radius of about 7 μm and tested it across a wavelength range of 450–785 nm. The reconstructed textures remained almost unchanged from blue to red (always close to 2), confirming broadband colored skyrmions from a single micro-crystal. The authors track the green output along propagation, showing that the structure is stable in propagation over many Rayleigh lengths. Importantly, the topological texture can be reconfigured by changing only the input polarization, offering wavelength versatility and tuneability in function.

This on-chip device advances prior bulky solutions^4,12^ in a single compact unit that requires no adjustment for wavelength tuneability yet has the versatility to alter the output texture by a simple polarization adjustment. While the initial advance is exciting, there remain open challenges. It is presently unable to alter the topological values and has so far been demonstrated only with classical light. The first may be addressed by materials engineering or cascaded devices, and intriguingly, the authors hint at a possible source of quantum states from this device too. If realized it may herald tuneable topological quantum states on-chip, a topic very much in its infancy yet promising exciting prospects for topological photonics by wavefunction engineering.

A few years ago, the topological toolkit was virtually empty, and open questions were posed as to the usefulness of optical topologies in general and skyrmions in particular. Today the state-of-the-art has advances to include creators, rudimentary detectors and the first switches for skyrmions. The present work shows how well-known forms of structured matter can be revisited and harnessed to make ever more sophisticated skyrmion devices, ushering in the essential tools to move optical topologies towards real applications.
